# Long-Term Outcomes of Chronic Cough Reduction after Laparoscopic Nissen Fundoplication—A Single-Center Study

**DOI:** 10.3390/medicina58010047

**Published:** 2021-12-28

**Authors:** Natalia Dowgiałło-Gornowicz, Anna Masiewicz, Justyna Kacperczyk, Paweł Lech, Sławomir Saluk, Karolina Osowiecka, Maciej Michalik

**Affiliations:** 1 Department of General, Minimally Invasive and Elderly Surgery, University of Warmia and Mazury, Niepodległosci 44 St., 10-045 Olsztyn, Poland; annalukuc@gmail.com (A.M.); justyna.kacperczyk@gmail.com (J.K.); lechpawel@op.pl (P.L.); slawomirsaluk@gmail.com (S.S.); 2 Department of Psychology and Sociology of Health and Public Health, School of Public Health, University of Warmia and Mazury, Warszawska 30 St., 11-041 Olsztyn, Poland; k.osowiecka86@gmail.com; 3 Department of General, Colorectal and Oncological Surgery, Nicolaus Copernicus University, Ujejskiego 75 St., 85-168 Bydgoszcz, Poland; michs1@wp.pl

**Keywords:** GERD, laparoscopic Nissen fundoplication, chronic cough, anti-reflux surgery

## Abstract

*Background and Objectives*: Gastroesophageal reflux disease (GERD) is one of the most common gastrointestinal diseases. It affects 20% of the adult population and is the third most common cause of chronic cough in adults. This study describes the results of LNF for the relief of GERD-related cough. *Materials and Methods*: The prospectively collected data on 135 laparoscopic LNF in our department from 2014 to 2018 were reviewed. During consultations, patients were asked about the frequency of symptoms using the GERD Impact Scale (GERD-IS), their satisfaction and recommendation to others, and their general condition after the procedure. *Results*: We analyzed 23 of 111 patients (20.7%) reporting chronic cough. The mean age was 47 years (range 27–76 years, ±13.9 years) and the mean follow-up time was 48.3 months (range 22.6–76.3 ± 18.05 months). Most patients reported relief from cough after the surgery (78.3%, *p* < 0.001). Five patients (22%) reported the recurrence of symptoms after a mean of 10.8 months (6–18 months). Seventeen patients (74%) would undergo the surgery again and 18 patients (78%) would recommend the surgery to their relatives. There was a statistically significant improvement in all symptoms from the GERD-IS (*p* < 0.05). *Conclusions*: LNF may play an important role in the management of GERD patients with extraesophageal symptoms. After LNF, most of the operated patients reported complete resolution of chronic cough and would recommend the procedure to their relatives.

## 1. Introduction

Gastroesophageal reflux disease (GERD) is one of the most common gastrointestinal disease. It affects 20% of the adult population [1]. It is a clinical manifestation of irritation or damage of esophageal mucosa. This is a result of abnormal reflux of gastric contents into the esophagus, caused by insufficient tension of the lower esophageal sphincter (LES) or hiatal hernia [2].

Typical symptoms include regurgitation and heartburn, often exacerbated by recumbency and heavy fatty meals [3]. In addition, GERD can manifest as extraesophageal symptoms, such as chronic cough, asthma, sore throat, laryngitis, and hoarseness [4]. These symptoms may occur with or without typical symptoms of GERD. As it turns out, GERD is the third most common cause of chronic cough in adults. GERD can stimulate the cough reflex through an direct or indirect mechanism. The first respiratory irritation, with or without aspiration. In the second mechanism, the symptom is produced by the esophageal tracheobronchial reflex via the vagus nerve [5]. Chronic cough is debilitating both physically and mentally. It leads to shortness of breath, sleep disturbances, and even urinary incontinence [6]. In addition, patients with chronic cough report fatigue, anxiety, and depression much more often [7]. This reduces the quality of life of patients [8,9]. Therefore, the reduction in chronic cough after surgery is an important factor for postoperative analysis.

The first-line treatment of GERD is always lifestyle changes, such as diet modification, weight loss, and improved sleep hygiene. Medical therapy is administered to patients with no noticeable improvement despite lifestyle changes. Medical therapy includes proton pomp inhibitors, antacids, and histamine 2 receptor antagonists [4]. The treatment that cures the cause of the disease, not just the symptoms, is surgical anti-reflux therapy. The most common method nowadays is laparoscopic Nissen fundoplication (LNF) [1]. Anti-reflux surgery seems to be an effective treatment of cough in patients with GERD [10]. The study describes the results of LNF for the relief of GERD-related cough.

## 2. Materials and Methods

### 2.1. Patient Selection

**Figure 1:**
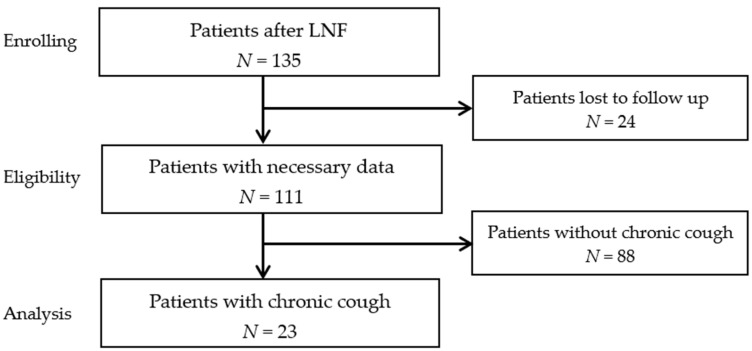
Flow chart of the study. LNF: laparoscopic Nissen fundoplication.

The prospectively collected data on 135 laparoscopic LNF in our department from 2014 to 2018 were reviewed. Twenty-four patients were lost to follow-up (follow-up rate 82.2%), and 23 patients were selected for the analysis due to chronic cough, Figure 1. The chronic cough has been assigned to GERD based on an extensive gastrointestinal and otorhinolaryngologic examination. The chronic cough was defined as a troublesome symptom of more than 3 month that the patient described in the questionnaire as daily or often (more than 3 times a week). Prior to surgery, all patients underwent objective examinations to establish the diagnosis of GERD, including esophagogastroduodenoscopy, 24 h pH monitoring, and otorhinolaryngological examination. The survey was performed before the operation, after 6 months, and once a year after LNF. During personal and telephonic consultations, patients were asked about the frequency of symptoms using the GERD Impact Scale (GERD-IS), their satisfaction measured by their recommendation to others, and their general condition after the procedure. GERD-IS consists of 9 questions that describe the frequency of the most common symptoms of GERD and their impact on everyday life. Due to the study’s design, it was not formally supervised by the Institutional Review Board in line with its policy.

### 2.2. Surgical Technique

Each patient underwent LNF according to the standard technique. After the dissection of the crura of diaphragm, the distal esophagus was mobilized approximately 5 cm. The crura were sewn sutured behind the esophagus with 2–3 non-absorbable sutures. A 360° posterior fundus wrap was constructed with 2–3 non-absorbable sutures.

### 2.3. Statistical Analysis

The descriptive statistics (mean, standard deviation, median, interquartile range) were estimated. The differences in GERD-IS before and after surgery were determined using Wilcoxon test. The differences between the subgroups: GERD-IS versus various factors were analyzed with the Mann–Whitney test. The comparison of the proportion in the subgroups was tested using the chi-square test. A *p*-value of <0.05 was considered to be significant. The analysis was conducted using STATISTICA software (version 13.3) (StatSoft, Krakow, Poland).

## 3. Results

In this study, we analyzed 23 of 111 patients (20.7%) reporting chronic cough. All patients underwent LNF in one surgical department from 2014 to 2018. Patients constituted 17 women and 6 men. Twelve patients (52%) reported daily, while 11 patients (48%) reported often coughs. The mean age was 47 years (range 27–76 years, ±13.9 years). The mean follow-up time was 48.3 months (range 22.6–76.3 ± 18.05 months). There were no postoperative complications in this group.

Most patients reported relief from cough after the surgery (78.3%, *p* < 0.001). Four patients (17%) required chronic administration of proton pomp inhibitors (more than one month after surgery). Five patients (22%) reported the recurrence of symptoms after a mean of 10.8 months (6–18 months). Seventeen patients (74%) would undergo the surgery again with their knowledge, while 3 patients (13%) would not do that. Three patients (13%) were unsure of responses. All patients who would not recommend LNF or were unsure of the response had recurrence of symptoms except one. Eighteen patients (78%) would recommend the surgery to their relatives, 3 (13%) would not do that. Two patients (9%) were unsure of responses. The recommendation was tantamount to satisfaction with the operation. All patients who would recommend LNF had no recurrence at the last follow-up survey, Table 1.

All patients were assessed with GERD-IS before and after the surgery. There was a statistically significant improvement in all symptoms from the questionnaire (*p* < 0.05), Table 2.

## 4. Discussion

A chronic cough is a serious problem for patients with GERD and has serious psychological and social consequences. According to the conducted studies, most people suffering from chronic cough described a negative impact on the quality of life, depression, social, and workplace shame [6,9]. Many patients complained of physical complications, such as chest pain, sleep disturbances, urinary incontinence, and vomiting [7,10]. Therefore, complete and lasting symptom relief has become an important point in the search for an effective treatment. Although guidelines for the management of typical GERD symptoms have been established, there are no specific data on atypical symptoms [11]. The role of anti-reflux surgery in improvement in extraesophageal symptoms in GERD is poorly understood, possibly due to diagnostic difficulties. Therefore, it is necessary to emphasize understanding of its atypical presentation and show doctors that effective treatment can be undertaken.

Our study reported the long-term results of LNF. In total, 20.7% of patients reported a cough as a daily or often symptom of GERD and most reported complete cough relief. The results of this prospective study suggest that LNF plays an important role in treatment of GERD patients with extraesophageal symptoms. We used GERD-IS in our study. It is a short nine-question questionnaire that describe the frequency of the most common symptoms of GERD and their consequences. This simple tool is useful in communicating with GERD patients, helping to objectify symptoms before and after GERD treatment [12,13].

Tutsumi et al. conducted a meta-analysis of the results of anti-reflux surgery in GERD respiratory symptoms [9]. Of the 61 analyzed studies, only 44% concerned Nissen fundoplication, the rest involved different surgical techniques or a combination of them. Some studies demonstrated open Nissen fundoplication. Regardless, cough relief affected 83.4% patients, which is similar to our results.

Park et al. presented one of the largest single-center studies in various surgeries, 89% of which was LNF [14]. They reported 77% of complete resolution of chronic cough and 71% of postoperative patient satisfaction. It is consistent with our data, 78% and 78% respectively. The reason for the dissatisfaction in our group of patients was the recurrence of symptoms, including cough, and the lack of surgery effect. This includes chronic use of PPIs. Similar conclusions are found in other reports [15,16,17,18,19]. Moreover, the patient’s mental state and possible mental illnesses have a significant impact on the patient’s satisfaction after anti-reflux surgery. Studies have shown that patients with anxiety show significantly less satisfaction with the procedure than the control group [20]. None of our patients had received psychiatric treatment or were taking psychiatric medications.

Several studies compared surgery with conservative treatment [21,22,23]. Surgeries are more effective than medical therapy. Zhang et al. compared proton pomp inhibitor therapy with LNF [24]. Eighteen patients with cough underwent LNF and 21 patients received esomeprazole 40 mg daily for approximately 3 months. After 2 years of follow-up, the LNF group represents a statistically significant improvement in symptoms and quality of life [24]. Antiacid drugs, such as proton pomp inhibitors cannot suppress other components of refluxate, such as bile, food, or enzymes; and esophageal irritation continues. Moreover, it is obvious that drugs cannot repair the hiatal hernia. However, not all patients with respiratory symptoms benefit from the surgery, therefore the decision to intervene should be made wisely and carefully. All other possible diagnoses of chronic cough should be excluded. The patient’s lifestyle and diet should be re-evaluated and GERD testing, such as 24 pH monitoring, must be performed completely.

This study has some limitations. The series is representative of a single, high-volume foregut center in Poland; however, the number of patients is limited. Nevertheless, the results of our work can be applied to the broad central European population. The other limitation is the loss of the patient during observation. After the first compulsory postoperative consultation, some patients did not reappear, which may indicate no complications or recurrence of symptoms, but unfortunately, we do not have the data.

## 5. Conclusions

LNF may play an important role in the management of GERD patients with extraesophageal symptoms. After LNF, most of the operated patients reported complete resolution of chronic cough and would recommend the procedure to their relatives.

## Figures and Tables

**Table 1:** Characteristics of patients after the surgery.

No	Chronic Use of PPIs	Undergo the Surgery Again	Recommendation of Surgery	Recurrence of Symptoms	Time of Recurrence [Months]
1	No	Yes	Yes	No	
2	No	Do not know	Do not know	Yes	12
3	Yes	Yes	Yes	No	
4	No	Yes	Yes	No	
5	No	Yes	Yes	No	
6	Yes	Do not know	No	Yes	12
7	No	Yes	Yes	No	
8	No	Yes	Yes	No	
9	No	Yes	Yes	No	
10	No	No	Do not know	Yes	18
11	No	Yes	Yes	No	
12	No	Yes	Yes	No	
13	No	Do not know	Yes	No	
14	Yes	Yes	Yes	No	
15	Yes	No	No	Yes	6
16	No	Yes	Yes	No	
17	No	Yes	Yes	No	
18	No	Yes	Yes	No	
19	No	Yes	Yes	No	
20	No	No	No	Yes	6
21	No	Yes	Yes	No	
22	No	Yes	Yes	No	
23	No	Yes	Yes	No	

PPIs: proton pomp inhibitors.

**Table 2:** Median and interquartile range of GERD-IS before and after the surgery. (1—daily; 2—often; 3—sometimes; 4—never).

Questions	Before LNF		After LNF		*p*
	Median	25–75% IQR	Median	25–75% IQR	
How often have you had pain in your chest or behind the breastbone?	2	1–3	4	3–4	<0.001
How often have you had a burning sensation in your chest or behind the breastbone?	2	1–3	4	3–4	<0.001
How often have you had regurgitation or an acid taste in your mouth?	2	1–3	4	3–4	<0.001
How often have you had pain or burning in your upper stomach?	3	1–4	4	3–4	0.03
How often have you had a sore throat or hoarseness that is related to your heartburn or acid reflux?	2	1–3	4	3–4	<0.001
How often have you had difficulty getting a good night’s sleep because of your symptoms?	2	1–4	4	3–4	<0.001
How often have your symptoms prevented you from eating or drinking any of the foods you like?	2	1–4	4	3–4	0.002
How frequently have your symptoms kept you from being fully productive in your job or daily activities?	2	1–3	4	4–4	<0.001
How often do you take additional medication other than what the physician old you to take (such as Maalox, Alusal, Manti)?	3	2–4	4	4–4	0.005

LNF: laparoscopic Nissen fundoplication; IQR: interquartile range.

## Data Availability

The data presented in this study are available on request from the corresponding author.
